# Defending the narrative self

**DOI:** 10.1098/rstb.2025.0155

**Published:** 2026-11-10

**Authors:** Marya Schechtman

**Affiliations:** Department of Philosophy, University of Illinois Chicago, Chicago, IL, USA

**Keywords:** self, identity, narrative, episodic memory, autobiographical memory, self-concept, social self

## Abstract

The idea that the self is narrative in form is widely expressed in both academic study and everyday speech. It has also been widely disparaged. Criticism from philosophers has been especially vociferous, arguing not only that narrative views do not represent the best conception of self, but that they are misleading and potentially dangerous. In this paper, I develop and defend an account of the narrative self that addresses these challenges. I begin by exploring the charges made against narrative views in more detail, uncovering three distinct but interrelated objections. Using lessons from constructivist theories of episodic memory and narrative accounts of the self in developmental psychology (especially that of Robyn Fivush), I describe a view according to which a particular form of self-narration constitutes us as mature human selves by conferring a range of cognitive, behavioural and experiential capacities that enable sophisticated forms of social engagement. On this view, the self is not constituted by a self-narrative so much as we constitute ourselves as human selves by becoming self-narrators who construct our narratives within the constraints of a social–cultural framework. I show how this account addresses each of the three objections outlined at the beginning.

This article is part of the discussion meeting issue ‘From being to identity: analysis and synthesis of the self’.

## 1. Introduction

The idea that the self is somehow narrative in form—that you *are* your story—has been widely expressed in both academic study and everyday speech. It has also been widely disparaged. Criticism from philosophers has been especially vociferous, arguing not only that narrative views do not represent the best conception of self, but that they are misleading and potentially dangerous. Our lives are not stories, critics insist, and narrative views are therefore necessarily both epistemically and morally harmful.

As is the case for most disputes that evoke passionate argument on both sides, there is a certain amount of talking past one another in debates about the narrative self. This paper aims to resolve some of the talking past and to articulate a view of the narrative approach that avoids the standard objections. Although critics offer plausible and familiar examples of potential pitfalls of a narrative conception of self, these are based on the assumptions that a narrative account of self must see our lives as like works of literature, constructed according to similar principles, and that whatever story we tell about ourselves is, according to narrative views, the unassailable truth of who we are. But this need not be so. We can see our lives as narrative in form without modelling them on literary narratives, and they can be narratives while still being constrained by fact. In what follows, I use resources from constructivist theories of episodic memory and developmental psychology to articulate an alternative picture of the narrative self. According to this view, we constitute ourselves as mature human selves by becoming self-narrators who track our ongoing lives within the constraints of a social–cultural framework.^1^

I begin (§2) with a discussion of the relevant objections to the narrative self. Next, I discuss how constructivist theories of episodic memory (§3) and narrative approaches in developmental psychology (§4) support a view of the narrative self as a natural developmental outcome for mature enculturated humans rather than, as critics seem to suppose, an optional, self-consciously constructed story we tell about ourselves. I then return to the original objections to the narrative approach (§5), showing how this account addresses them.

## 2. Objections to the narrative self

A common line of criticism levelled at narrative approaches to self argues that narrative is a singularly inappropriate framework for understanding the self. Narratives, critics argue, are inherently situational and selective, and this means that no narrative of a human life can possibly represent it accurately. Self-narratives, which are often taken to constitute the self according to narrative views, are especially suspect. The events we include in our self-narratives and our sense of their significance, critics warn, are inevitably shaped by distorting motivations—self-interest making them self-aggrandizing, or coercive social pressures making them oppressive. Trying to fit our lives into a narrative structure, they worry, makes us misrepresent our histories. Peter Goldie, for instance, says that ‘the demands of narrativity … seem to drag us towards thinking of our past thoughts, feelings, and deliberations as more determinate than they in fact were, and as reflective of an agency of which at the time we seemed quite bereft’ [1]. Galen Strawson argues that narrative always involves revision and invention. Turning our lives into narratives, he says, overwrites what actually happened, replacing it with a fictional version. In self-narration, one therefore risks moving away from ‘accurate self-understanding’ and the ‘truth of one's being’ [2]. Self-narrative necessarily distorts the self, according to Peter Lamarque, because literature and life are inherently different kinds of endeavours. Novels are artefacts created for aesthetic purposes, with each event and word chosen to further those purposes. Lives, by contrast, are messy natural processes, filled with accident and contingency [3].

The harms of mistaking one's self-narrative for the actual self are not just epistemic, critics emphasize. The self-deception and self-aggrandizement/deprecation associated with self-narrative also interfere with wellbeing and moral engagement. Our tendency to remember ourselves as better, smarter, kinder and braver than we were prevents us from recognizing and addressing character flaws. Alternatively, we might take up a pathologically negative narrative that prevents us from recognizing our own worth. As Galen Strawson puts it, narrative understandings of the self can ‘close down important avenues of thought, impoverish our grasp of ethical possibilities, needlessly and wrongly distress those who do not fit their model and are potentially destructive in psychotherapeutic contexts’ [2, p. 429].

Many of the worries raised about narrative seem plausible, but to be clear on their implications we will need to think more carefully about what the objections really claim and what evidence is provided for them. On close inspection, there seem to be three main, interrelated but distinguishable objections levelled against the narrative self. One (seen in Lamarque's objections [3]) is a broad rejection of narrative as a vehicle for self-understanding or self-constitution based on the disconnect in form between narrative and human life. Selves are not stories, according to this objection, and presenting them as such necessarily distorts them. A second, somewhat more modest claim (of which Goldie's is an example [1]) is that self-narratives are subject to irresistible distorting influences. We are motivated to see ourselves in a good light or play down our faults, and our sense of self can be infiltrated by pernicious stereotypes. The claim here is not about the narrative form itself, but the inherent unreliability of self-narratives as examples of the form. The third claim (seen in Strawson [2]) is the practical upshot of the first two. It argues that it is a harmful mistake to think of oneself as living a story and that we would do well to break ourselves of this habit.

The forms of unreliability and distortion these critics describe are familiar and plausible enough, but the conclusion that they negate the possibility of an illuminating and valuable narrative conception of self rests on implicit assumptions about epistemology and about the role narrative plays in constituting the self. The next two sections provide the resources to challenge these assumptions and develop an alternative understanding of the narrative self.

## 3. Constructivist views of episodic memory

In arguing that the form of narrative necessarily distorts the truth of the self, critics tend to point especially to three features: first, narrative is by its nature *selective*. In developing a life narrative, we include some elements of our personal histories and not others. Second, it is *interpretive*. Narrative does not provide an objective recapitulation of historical facts but puts a spin on what we remember. Third, narrative is *reconstructive*. We remember in ways that serve our narratives rather than as things really happened. Because of this, narratives will inherently omit parts of our histories that would cast us in a different light, fail to be objective and deviate from fact. This means, critics conclude, that they cannot help but misrepresent the truth of the self.^2^

I concede that narrative, self-narrative included, is selective, interpretive and reconstructive, but I reject the conclusion that this necessarily falsifies the self or makes narrative understandings of self harmful or misleading. If self-narrative were to select, interpret and reconstruct in just the way an author does in crafting a literary work, the resulting presentation of the self would indeed be suspect. But there are other principles by which we can and do select, interpret and reconstruct information that are both illuminating and valuable. This section uses constructivist views of episodic memory^3^ to support this general claim. The next section will provide more details about how the lessons learned here apply specifically to self-narrative.

The most straightforward way in which narrative mechanisms might appear falsifying is in their reconstruction of the personal past that represents what happened in a way that demonstrably deviates from the facts. My memory that I made a brilliant comment in a faculty meeting or took a heroic action last week may fit nicely into my self-narrative, according to which I am brave and wise. If in fact it was my colleague who made the remark or my neighbour who undertook the heroic deed, however, my self-narrative seems simply false. If we understand ‘truth’ as faithful correspondence to historical fact, and falsity as the opposite of truth, this argument is sound.^4^ Whatever our epistemological commitments, however, the more important point here is that deviating from historical fact does not always make a narrative misleading or harmful. On the contrary, there is reason to think that some forms of memory reconstruction yield insights more valuable and illuminating than strict reproduction.

The fact that our memories of specific past events often get details wrong is not news to anyone. I may vividly remember stopping at that diner with the great pie on our last road trip, but misremember the colour of the rental car we were driving, or the song playing on the jukebox when we walked in. The frequency of these common kinds of mistakes is empirically well documented [10]. If deviation from a comprehensive and entirely faithful reproduction of an event in one's history in recalling the personal past is a problem as critics allege, it is one that applies more broadly and not just to narrative views. Some forms of misremembering are highly pathological, of course, and even trivial forms of mistaken memory can have ill effects in specific contexts (e.g. eyewitness testimony in court). A crucial lesson of recent memory research, however, is that common misremembering is not always problematic, but can be instead adaptive.^5^

The idea that memory is reconstructive, and that this is not necessarily a bad thing, is not new. In 1932, Frederic Bartlett argued that the nature and ubiquity of common memory errors suggest that we must give up the idea ‘that memory is primarily or literally reduplicative, or reproductive. In a world of constantly changing environment, literal recall is extraordinarily unimportant … memory appears to be far more decisively an affair of construction rather than one of mere reproduction’ [13, pp. 204–205]. Recent decades have yielded deeper understanding of the cognitive and neural processes by which memory reconstruction takes place [14–17]. A strong connection between episodic memory and imagination has been demonstrated both in clinical contexts (patients with deficits in episodic memory also show difficulty imagining future or counterfactual scenarios [8,18]) and in neuroimaging studies, which show significant overlap in the neural mechanisms involved in episodic memory and those involved in imagining future and counterfactual scenarios [14,16,17,19]. This has led to the suggestion that instead of a dedicated system for reproducing past experiences in episodic memory, we have a more general system for constructing representations of specific episodes or scenes [20;21, pp. 100–101]. This system can produce episodic memories, representations of past counterfactuals (episodes that might have occurred but did not), or imaginings of possible future episodes.

These findings support Bartlett's original assertion that memory errors in healthy people can be adaptive. Schacter and Addis [16,22,23] argue that, in many cases, alterations of an experience in memory are not the result of random error. They occur instead because, during the process of reconstruction, the event remembered is interpreted, contextualized and updated in ways that are cognitively useful. Schacter [24] concludes that the ‘seven sins’ of memory (transience, absent-mindedness, blocking, suggestibility, bias, persistence and misattribution) that are commonly found in healthy populations serve an adaptive purpose. These manipulations allow us to extract relevant information from past experiences and present it in a way that serves present needs efficiently. Bartlett suggested that the memory errors he observed ‘were based on the operation of a schema that serves to organize and interpret experiences’ for our use, and Schacter sees present findings as vindicating this insight [13,15].

De Brigard emphasizes that this adaptive function of memory reconstruction undermines standard philosophical assumptions about the epistemology of memory. In the provocatively titled ‘Is memory for remembering?’ [25], he notes that it is often taken for granted in philosophy that an ideally functioning memory system should faithfully reproduce past experiences. Although it is acknowledged that we misremember all the time, this is taken to be an unwanted consequence of the imperfection of our memory systems. The findings of Schacter and Addis, De Brigard argues, show that, on the contrary, we are frequently better off with a representation of an episode that presents information that has been condensed and distilled from multiple relevantly similar past experiences than we would be with an accurate recreation of one specific experience. Perfect fidelity should not always be taken as the ideal.

If we accept this approach, the argument that reconstruction by itself makes self-narrative misleading is not compelling. The kind of reconstruction I have described here involves selection and interpretation, so these narrative elements are also vindicated in this context. The narrative form thus does not seem necessarily distorting. It remains possible, however, that reconstruction is a special problem in the context of self-narrative. The kind of reconstruction that takes place in episodic memory according to constructivist views occurs at the sub-personal level as the operation of an evolved episodic memory system that serves to provide effective information about how to navigate the natural environment. Self-narration, by contrast, is a higher-order, linguistically mediated form of reconstruction that provides information about the self. To assess the value of reconstruction in the narrative context, I turn to work in developmental psychology.

## 4. Autobiographical memory and the narrative self

Narrative views of self have played an important role in developmental psychology [26–32]. These views hold that humans construct an internalized, evolving life story, a process that begins in the preschool years and continues throughout the life course. This self-narrative provides a sense of oneself as a continuing being and generates a suite of capacities that allow new kinds of engagement with the natural and social world. Understanding these views better provides insight into how and when selection, interpretation and reconstruction can make a self-narrative illuminating and valuable, and what principles must be followed for it to do so.

Although there are many narrative theorists, Robyn Fivush puts special emphasis on how the reconstructive resources of episodic memory are harnessed and developed to produce a self-narrative, and I will focus on her work here. Especially important for our purposes is a distinction Fivush draws between episodic memory and what she calls ‘autobiographical’ memory [33].^6^ Tulving's [8] definition of episodic memory includes two components. One is ‘the idea that the memory is specific to what, when, and where (i.e., that the memory is of a specific event that happened at a particular time and place in the past)’ [p. 562]. The other is a sense of ‘mental time travel’, a feeling that we are ‘revisiting’ the past. This involves autonoetic awareness of the episode remembered as part of one's personal history. Fivush believes it is a mistake to assume that these two features always occur together (see also [34]).

There is ample evidence, she argues, that ‘what, where, when’ representation can be present without autonoesis. Research on the question of whether nonhuman animals have episodic memory ‘clearly demonstrates … that animals can recall specific experiences that occurred in the past and can use these experiences to guide their current and future behavior’. Moreover, the ability to remember specific past experiences is also demonstrated in the first year of human life, before autonoetic awareness is present [33, p. 562]. She therefore distinguishes ‘episodic memory’, the kind of ‘what, when, where’ memory found in young humans and nonhuman animals, from the ‘ability to represent oneself as an experiencer of events, as a continuous being with a past, present, and future that links specific episodic representations into a meaningful sequence of events that define a person and a life’, which she sees as ‘a uniquely human ability’ [33, p. 563]. ‘Autobiographical memory’, as she defines it, is this ‘uniquely human form of memory that moves beyond recall of experienced events to integrate perspective, interpretation and evaluation across self, other and time to create a personal history’ [33, p. 560].

This kind of autobiographical memory is an acquired form of memory, a ‘complex human skill’ that ‘continues to develop and evolve across the lifespan’ [33, p. 560]. It is, moreover, a skill that requires intensive social scaffolding in its development. Children need to be taught how to structure their memories in complex narrative forms by ‘participating in socially and culturally organized reminiscing in which one's own memories of a past event can be compared to another's.’ These interactions provide children with ‘the skills for creating more coherent and connected narratives that lead to the construction of a personal timeline’, thereby developing autonoetic consciousness, or ‘the sense of a subjective self as an experiencer of events’ [33, p. 565].

As carers rehearse the day's events with children, often at bedtime, they help them develop expressions of recalled events into rudimentary stories that connect these events to other events, to affect, to their consequences and to future plans. If the child says ‘strawberries’, for instance, suggesting a recollection of eating strawberries earlier, the carer might reply, ‘Yes, we had strawberries today, they were yummy weren't they?’, adding, perhaps ‘Where did we get the strawberries? That's right, we went to the store and got them and brought them home. We ate them while we watched your show. What happened when your brother came home? That's right, you wouldn't share so I took the strawberries away, didn't I? Your brother was sad, wasn't he? But then you said ‘sorry’ and made up. What do you think? Do you want strawberries on your birthday cake?’ When regularly exposed to this kind of reminiscence (and projection), children begin telling simple narratives on their own. These stories become increasingly complex over time, turning eventually into mature adult narratives. As self-narratives become more sophisticated and robust, so does the subjective sense of self, which constitutes both a sense of identity through time and the autonoetic awareness that characterizes autobiographical memory. ‘Whereas episodic memories are of a series of single past events’, Fivush says, ‘autobiographical memory links past events together into a personal history that relates self through past, present, and future, essentially forming a life narrative’ [33, p. 560].

The representational structure and content of simple ‘what, when, where’ memory is altered by subsuming it into a narrative. Personal memory in adults is complex, often including not only easily demarcated, discrete events but also extended events (e.g. a vacation to Paris), repeated events (e.g. birthdays when I was growing up) and life periods (e.g. while I was at university) [35,36]. Fivush follows the changes in content and structure of personal memories in Nelson's [37] recordings of 2-year-old Emily's bedtime conversations with her parents and pre-sleep monologues over a 15-month period. At the beginning, Emily recalls very specific events of short duration. Over time, she describes events spanning longer and longer durations. Eventually, she starts to embed descriptions of subroutines within her recollection of routines (e.g. eating breakfast as part of the routine of getting ready to go to preschool), includes more mention of her own thoughts and feelings, talks more about the thoughts and feelings of others and what they are likely to do, and connects these causally. Her recollections become increasingly narrative in form, more diachronically extended and hierarchically organized, developing ‘from a loose collection of memories that are not coherently organized in time to recollection of related memories organized along a timeline’ [38, p. 279].

Autobiographical memory is not, as Fivush sees it, a separate system that replaces episodic memory, but rather an augmentation of it.^7^ She takes on board the basic constructivist picture of episodic memory as involving adaptive reconstruction that allows us to navigate the natural world efficiently (described in §3), and argues that this purpose can be met without autonoetic awareness. Autobiographical memory, however, serves a different purpose, which does require autonoesis. ‘Whereas event memory serves the function of organizing our knowledge of the world, a predictive function’, she says, ‘autobiographical memory serves the function of organizing our knowledge about ourselves, a self-defining function’ [38, p. 277]. It ‘goes beyond the episodic memory function of guiding current and future behaviour to serve social and emotional functions’ [33, pp. 560–561]. For this reason and based on ‘findings that individual autobiographical memory is socially and culturally variable and dependent on specific social-cultural interactions’, she speculates that this form of memory developed in humans to serve primarily social and cultural purposes [33, p. 574].

Although this gives autobiographical memory a different set of reconstructive principles from episodic memory (organizing, contextualizing and updating information about the self for purposes of social interaction as opposed to organizing, contextualizing and updating information about the natural world to navigate it more effectively), Fivush is explicit that these are not separate memory systems. The ‘same memories’, she says, can simultaneously allow us to anticipate and predict the world around us and help define how we think about ourselves’ [38, p. 277]. If episodic memory condenses, interprets, contextualizes and updates information about the world gained from multiple experiences, autobiographical memory condenses, interprets, contextualizes and updates information about the *self* over time. In so doing, it adds a new dimension to our overall memorial structure, making reconstructive use of the fully narrative tools of social–cultural schemas in addition to the proto-narrative reconstructive tools of the episodic memory system.^8^

The narrative organization found in autobiographical memory requires language. ‘Narratives are canonical linguistic forms that specify a sequence of actions and the links between them’, Fivush says. But autobiographical memory is not itself linguistic [33, p. 565]. Like all memory, she continues, ‘autobiographical memory is composed of multiple sensory components that are stored and retrieved across multiple brain areas [36,39]. … Thus, the argument is that culturally defined narratives, linguistic forms that provide schemes and guidelines for understanding how human events occur [40] help shape the way in which individuals come to recall their personal past’ [33, p. 565]. Shared reminiscing adds new reconstructive tools to the episodic toolbox in the form of socio-cultural norms, causing us to remember in new ways. The complex form of memory this yields simultaneously expresses useful information about the natural world and about oneself in the social world, allowing us to predict and act effectively in each domain.

It is worth pointing out, though this is not something Fivush emphasizes, that social reminiscing does not just teach children how to organize specific memories, connecting them to one another, and to their social circumstances. It also conveys an abstract overall template for living a human life and encourages the child to internalize a sense of itself as a human among humans, living such a life.^9^ In social reminiscing, children are prompted not only to look backward and organize their understanding of what happened but also to consider what might have happened, to connect their understanding of the actual and counterfactual past to how things are for them now, and to use this understanding to anticipate and plan for the future. As the span of autobiographical memory expands, so does the span of past and future projection, and of overall narrative scope scaffolded by carers. Children are encouraged to look forward to when they will go to school like their elder siblings or get married and have children of their own. They are asked what they are going to be when they grow up. The specifics of these promptings vary in different social–cultural contexts. Across cultures, however, children are made aware of the standard cultural milestones and know roughly the shape their own life is expected to take [41,42]. As Addis *et al.* put it, we ‘possess an understanding of how, within our culture, the prototypical life should unfold in terms of the order and timing of significant life events’. This, they say, provides a ‘temporal ‘template’ that organizes past experiences and gives us a sense of what to anticipate in the future’ [35, p. 800;^40^,^41^].

This does not mean that everyone will automatically meet every cultural milestone, nor that they will, or should, aspire to. Nonetheless, these milestones, met or missed, pursued or rejected, form a backdrop against which our day-to-day experience takes place. It is not just *what* happened in the past that influences our experience or actions, but *when* it happens. Setbacks when one is starting out in adult life, for instance, may have a different valance from those that occur towards its end. A medical complication or a windfall may similarly have different significance depending on when it occurs, in part because this affects the degree to which it interferes with or supports the expected unfolding of a life [43,44]. In addition to tracking and organizing the specific details of our lives and their relation to one another, self-narration also tracks progression along the life course. This tracking is also usually implicit, operating in the background. I know roughly where I am on my life course, whether I am just starting out or nearing the end, whether milestones like finding a mate, having children, building a career (or personally and culturally appropriate analogues) are probably in front of or behind me. This all makes a difference in how I see and move through the world.

In the typical case, the tracking of specific autobiographical facts and of whole life progress are deeply interconnected. Just how much the development of autobiographical memory alters our experience of self-in-time can be seen, however, by noting that the overall sense of oneself living a human life appears capable of surviving the loss of the capacity for autobiographical memory that gave rise to it. Addis and colleagues have found that individuals with Alzheimer's disease (AD) appear, at least for a time, to retain perception of themselves as diachronically unified individuals leading a human life, despite severe deficits in autobiographical memory [35,45]. Studies on individuals with episodic amnesia also reveal that their attitudes towards their own pasts and futures, including temporal discounting and the ability to predict future regret, remain intact [46,47]. Those with severely compromised autobiographical memory often express distress at their inability to place and contextualize their experience. The experience of lack seems to presuppose retained awareness that they are living a life that is theirs to track, while lacking the wherewithal to do it.^10^

The understanding of the narrative self I propose is thus a self that emerges from a more basic or ‘minimal’ self (e.g. [48]) when children are inducted into the practice of self-narrative within their cultural context, coming to track their progress along a broad expected life trajectory, both implicitly and explicitly, at many levels of specificity and awareness. This tracking gives rise to experiential, emotional and cognitive capacities, which in turn make it possible to engage in activities, abilities and forms of experience that characterize the mature human self. These include, for instance, developing and pursuing long-term goals and plans, considering the future consequences of acting on our desires, experiencing existential angst and committing to and fighting for our values. A self-narrative, on this view, is not a story I might or might not decide to spin about myself. It is a way of organizing experience that serves to constitute a mature human self, and its effects are profound and transformative.

## 5. Answering the objections

Section 2 described three interrelated objections commonly levelled against narrative approaches to the self. Having outlined the particular narrative view I wish to defend, I am now in a position to offer a quick account of how it can respond to each objection.

The first objection targets narrative form, arguing that self-narration is necessarily misleading (and probably harmful) because narrative is inherently selective, interpretive and reconstructive. The discussion of constructivist accounts of episodic memory in §3 gave us reason to reject the assumption that selection, interpretation and reconstruction automatically mislead. Memory reconstruction that summarizes and contextualizes information gained in the past, presenting it in a way that informs effective prediction and interaction with the environment is, to the contrary, illuminating and beneficial. Fivush's account of self-narrative as a means of organizing experience in a way that facilitates social coordination and engagement shows how selection, interpretation and reconstruction are similarly instructive and useful in the context of self-narration insofar as they succeed in presenting summarized and contextualized information about oneself that supports effective social interaction.

The second worry concerns biases that, according to critics, distort self-narratives. Even if a self-narrative could in principle be illuminating, this argument suggests, in practice it will not be because of these powerful motivations to narrate inaccurately. I do not deny that such biases exist, nor that they can and do sometimes mislead us. But this does not mean that they will inevitably do so, or even that they usually do. The examples of motivated self-misrepresentation provided are anecdotal, and we are given no information about their frequency. Moreover, it is not obvious that, even when they do occur, they make one's self-narrative deeply misleading. Just as many deviations from historical fact in episodic memory are trivial and harmless, so are many minor distortions in self-narrative. Moreover, the view I have described provides a criterion for identifying and potentially correcting significantly deviant self-narratives.

Once again, an analogy with episodic memory is useful. When memory is reconstructed in a way that is not suited to its purpose, it will lead to failures of prediction and ineffective interactions with the environment. If this happens only occasionally, it can provide important information that can be used to improve reconstructive success in the future. Reconstruction that persistently and pervasively leads to ineffective interactions with the environment, however, is pathological, and we have a principled way to identify it as such. To say that reconstructed memory is not necessarily misleading just because it deviates from faithful reproduction of historical fact is not to say that it is *never* misleading. The relevant point here, however, is that this is the exception rather than the rule. The existence of occasional pathology does not undermine the claim that reconstruction done well can be instructive and valuable, even if (indeed because) it deviates from faithful reproduction of a past event.

I suggest that the situation is much the same for self-narrative. In many cases, failure to faithfully reproduce past events in autobiographical memory will not be importantly misleading. If, for instance, I remember making a particular brilliant remark that I did not, but I make such remarks often and am widely known for my brilliance, the fact that I was wrong about this instance does not make my narrative fail to represent my nature, and will probably have few implications for social interaction. If, on the other hand, I consider myself generous because I have selectively included my few acts of largesse in my narrative, leaving out the vastly more numerous occasions on which I was withholding and stingy, the situation is different. Here, I will be operating with a sense of myself that will probably lead to false predictions about how I will be received by others and otherwise interfere with effective social interaction. In such cases, I will receive pushback from the social world in something like the way faulty episodic reconstruction leads to pushback from the natural environment. This might lead me to reconsider my self-narrative, adjusting and updating it. If this feedback does not have such an effect, however, and I persistently and pervasively select, interpret and reconstruct in ways that are deviant, my narrative will fail in its purpose of facilitating effective social interaction, and we will be able to identify it as pathological.

That this is so, is implied by the objection itself. The case that self-narrative is unreliable because it is subject to distorting biases is supported through examples. To do the intended work, the examples given must be clear and uncontroversial instances in which events are selected, interpreted or reconstructed in a way that results in a misleading or false self-narrative. Otherwise, they would fail as compelling examples of a falsifying narrative. If the faultiness of these acts of self-narration is evident in the examples, however, it will also be evident if we encounter such a case *in vivo*. We do meet people who we can recognize as unreliable self-narrators, impervious to feedback. And probably many, or even most, of us have biases or motivations that slightly distort our own sense of self. But the kind of systematic bias that fundamentally misrepresents who someone is in a way that constitutes or borders on delusion is relatively rare. The purpose of self-narrative, according to my view, is not to provide an entirely faithful version of one's history, or a completely unassailable representation of one's nature. It is to engage effectively in the social world.^11^ Small detectable distortions are compatible with this purpose, and pervasive and significant distortions, which are not, are recognizable as pathologies.

The third objection suggests that we would be better off if we were not self-narrators. It is difficult to answer this charge directly because it seems aimed at a narrative view according to which we decide to make our lives conform to some particular narrative archetype taken, perhaps, from myth, or literature, or film. This is very different from the view I have proposed, in which we acquire the capacity to constitute ourselves as mature human selves by learning to organize our experience narratively, tracking our ongoing lives in ways that allow us to make ourselves intelligible to others and to ourselves. In this picture, self-narration is not something we choose, but something that emerges as a developmental stage of humans in culture. Asking whether we *should* organize our experience in this way is somewhat like asking whether we should be episodic rememberers. Perhaps, because self-narration is an acquired capacity, there is a possibility for choice with respect to being self-narrators that is not present with respect to an evolved biological system like episodic memory. But the very ability to raise this question requires that we conceive ourselves as temporally extended beings who care about how our lives go and wonder about what kind of social organization will make them go best. To conceive and consider it, we need the kinds of cognitive tools bestowed by self-narration. Even if we could reject this form of self-narration, doing so would make us quite other kinds of beings than we are.

## 6. Conclusion

The standard objections raised against narrative conceptions of the self are aimed most directly at the view that we spin stories about who we are and what we are like, and that whatever story we spin is, by definition, the truth about us. I agree that such a view has serious difficulties, but it is not the only way to think of the narrative self, and I have offered an alternative. Beneath the specifics of these objections, however, there is an underlying thought that suggests a broader challenge that any narrative view does need to meet. At the most general level, the standard objections express a worry that if the self is narrative and narratives are stories we make up, there is no room for any self-narrative to be false or delusional, and it seems obvious that sometimes they are. I accept the claim that self-narratives can be, and sometimes are, misguided or delusional, but reject the assumption that seeing the self as a narrative construct makes this inevitable or even common. All that is required to avoid it is for there to be non-arbitrary constraints on identity-constituting self-narration. My disagreement with the critics is about what these constraints are and where they come from.

The standard objections imply that the only acceptable constraint is a strict and faithful correspondence to historical facts. This approach is implausible, suggesting that an accurate sense of self would have to be a comprehensive and uninterpreted representation of all the events in our history. Constructivist views of episodic memory provide a model of how successful construction can instead be constrained by success relative to a purpose, and Fivush's social-developmental view of autobiographical memory shows how this insight can be applied to self-narratives, which serve the purpose of allowing effective social engagement. We cannot make up just any story about ourselves and have it be true. But there are different ways a self-narrative can go wrong. Here are two: it can deviate from historical fact, or it can fail to be an accurate representation of what we are like as participants in the social world. I suggest that the constraints needed to avoid the second sort of failure are possibly more important. These come from the social world, and to meet them we need to be able to track our lives within basic social–cultural constraints. This is just what our self-narratives allow us to do, constituting us as mature human selves, even if we sometimes get things wrong.

## Data Availability

This article has no additional data.

## Footnotes

^1^
As I hope will become evident, I do not say that these are the only constraints on our memories. I do want to say, however, that they are the ones most relevant to defending the narrative self and the ones that are overlooked by its critics. Nor do I mean to imply that self-narration is the only contributor to mature human selfhood, only that it is a critical part of the process in ways to be described later in this paper.

^2^
An anonymous referee very helpfully pointed out that there is a fourth objection also raised against narrative views, and this is their claim of universality. Some critics (especially [2]) argue that only some humans have narrative self-experience and that narrative is unnecessary for a distinctively human life. I think that there is some truth to this objection, but that it overstates the case. Limitations of space prohibit me from addressing it here, but the heart of the response can be found in the discussion of §4, which will show the sense in which I think narrative has a claim to universality. I also speak to this question directly in [4,5].

^3^
Episodic memories are memories of specific events in the personal past, usually involving imagery and/or affect and entailing a sense of ‘reliving’ or ‘revisiting’ an experience and an awareness of the event remembered as one we experienced (autonoetic awareness) [6–8]. I will discuss these components in more detail as I proceed.

^4^
It is worth noting, however, that this is not the only available account of what it is for something to be true [9].

^5^
This is not to say that reconstructed memories cannot ever be faithful reproductions of past experiences. Indeed, one would expect that memory is often faithful to past events in many respects. See, for example [11,12]. The points I wish to make here are that it is also common for memories not to be exact and faithful reproductions of the past in all respects, that perfectly faithful reproduction should not be assumed to be the relevant ideal, and that deviation from faithful reproduction is not always a deficit.

^6^
Not everyone accepts this as a legitimate distinction and, even those who do, do not necessarily define episodic and autobiographical memory as Fivush does. My concern here is with the phenomenon itself, whatever we call it, so I will put that controversy to one side.

^7^
See [35] for a related (non-developmental) analysis of diachronic self-experience and its relation to more punctuated episodic memory.

^8^
For related discussion of the importance of social–cultural schemas to autobiographical memory and sense of self see [35].

^9^
This abstract ‘life template’ is a background feature of the embedded narratives of shorter spans of time that are organized within it. I see it as the culmination of the development of increasingly complex, extended and hierarchical forms of narrative organization described in the reflections on Emily above.

^10^
The issues here are of course complicated. The sense of oneself as living a life I am describing is not always retained in AD or amnesia. In most cases where it is retained, some semantic memory of one's history is present. Even when the sense of one's own continuity is retained through loss of autobiographical memory, moreover, it is not unaffected. My point is only that those cases in which a sense of oneself living a human life—an overall narrative structure—is retained through the loss of autobiographical memory are sufficient to show that this is a distinct feature of the narrative self, above and beyond tracking the specific details of an ongoing life story.

^11^
It might, of course, serve other purposes as well, for instance, allowing us to recall more detail than we would otherwise be able to retain. But here I am focusing on its identity-constituting function.

## Theme

One contribution of 17 to a discussion meeting issue ‘From being to identity: analysis and synthesis of the self’.
